# Developing a standardized healthcare cost data warehouse

**DOI:** 10.1186/s12913-017-2327-8

**Published:** 2017-06-12

**Authors:** Sue L. Visscher, James M. Naessens, Barbara P. Yawn, Megan S. Reinalda, Stephanie S. Anderson, Bijan J. Borah

**Affiliations:** 1 Robert D. and Patricia E. Kern Center for the Science of Health Care Delivery, Mayo Clinic, 200 First St SW, Rochester, MN 55905 USA; 2 Division of Health Care Policy and Research, Mayo Clinic, 200 First St SW, Rochester, MN 55905 USA; 3 Department of Research, Olmsted Medical Center, 210 9th Street SE, Rochester, MN 55904 USA; 4 Division of Biomedical Statistics and Informatics, Mayo Clinic, 200 First St SW, Rochester, MN 55905 USA

**Keywords:** Cost data warehouse, Standardized healthcare costs, Microcosting, Olmsted County Healthcare Expenditure and Utilization Database (OCHEUD), Rochester Epidemiology Project (REP)

## Abstract

**Background:**

Research addressing value in healthcare requires a measure of cost. While there are many sources and types of cost data, each has strengths and weaknesses. Many researchers appear to create study-specific cost datasets, but the explanations of their costing methodologies are not always clear, causing their results to be difficult to interpret. Our solution, described in this paper, was to use widely accepted costing methodologies to create a service-level, standardized healthcare cost data warehouse from an institutional perspective that includes all professional and hospital-billed services for our patients.

**Methods:**

The warehouse is based on a National Institutes of Research–funded research infrastructure containing the linked health records and medical care administrative data of two healthcare providers and their affiliated hospitals. Since all patients are identified in the data warehouse, their costs can be linked to other systems and databases, such as electronic health records, tumor registries, and disease or treatment registries.

**Results:**

We describe the two institutions’ administrative source data; the reference files, which include Medicare fee schedules and cost reports; the process of creating standardized costs; and the warehouse structure. The costing algorithm can create inflation-adjusted standardized costs at the service line level for defined study cohorts on request.

**Conclusion:**

The resulting standardized costs contained in the data warehouse can be used to create detailed, bottom-up analyses of professional and facility costs of procedures, medical conditions, and patient care cycles without revealing business-sensitive information.

After its creation, a standardized cost data warehouse is relatively easy to maintain and can be expanded to include data from other providers. Individual investigators who may not have sufficient knowledge about administrative data do not have to try to create their own standardized costs on a project-by-project basis because our data warehouse generates standardized costs for defined cohorts upon request.

**Electronic supplementary material:**

The online version of this article (doi:10.1186/s12913-017-2327-8) contains supplementary material, which is available to authorized users.

## Background

A challenge for researchers addressing value-based healthcare is how to measure cost for public dissemination of research results [1]. There are many types of costs; the most commonly used are direct healthcare costs (e.g. professional services, supplies, medical facilities).

Direct healthcare costs are obtainable from many different sources. Information about charges may be available publicly, but charges do not accurately reflect either provider or payer cost because of markups and discounts [2]. Actual reimbursement is a useful cost measure, but many providers do not have data systems that link payments to specific services, and those who do also need to be aware of antitrust or payer contract issues when the information is disclosed [3, 4]. Reimbursement in the form of claims data from payers has limitations as well. Medicare and Medicaid claims data cover only segments of the population. In addition, Medicare and Medicaid use a prospective payment system for facility costs that apply fixed payments to groups of services. For example, the effect of length of stay on cost cannot be measured. Commercial insurance claims, including actual reimbursement, are usually de-identified with respect to patients and providers. In addition, different contract terms produce different unit costs for providers of the same service [5].

Internal costs are the most useful measure for providers to evaluate cost drivers and increased efficiencies, but not all providers have complete cost-accounting systems [6]. Furthermore, many providers consider these internal costs to be business sensitive and do not want them shared publicly. Although proportions and percent changes in actual costs might be published, results can be difficult to interpret without a dollar frame of reference. Finally, many authors appear to create their own study- specific cost datasets based on a mixture of these cost types, although this process can be expensive [5]. The explanations of their costing methodologies are not always clear, so their findings may be difficult to interpret [7].

This paper describes our solution, which was to create a standardized cost data warehouse that uses accepted methodology [8] to assign a relative dollar value of resources to every billed professional and hospital service provided to every patient (microcosting). We demonstrate how investigators can create a cost data warehouse that can be used to support detailed, bottom-up analyses of professional and facility costs of procedures, medical conditions, and patient care cycles without revealing business-sensitive information.

## Methods

### Source data

We have two cost data warehouses that share the same reference file and programming code infrastructure: one for the National Institutes of Health–funded Rochester Epidemiology Project (REP) and one for Mayo Clinic in Rochester. Both were formerly known as the *Olmsted County Healthcare Expenditure and Utilization Database*. The REP warehouse is affiliated with the larger REP [9, 10], which is a research infrastructure containing the linked health records and medical care administrative data of virtually all persons residing in our county. An electronic data-sharing agreement was signed by the leaders of Mayo Clinic campus in Rochester, Minnesota, and the Olmsted Medical Center (OMC) in 1995 for the purpose of sharing and archiving patient-level administrative data on healthcare utilization and the associated direct medical costs of care.

The REP warehouse uses billing data from Mayo Clinic in Rochester and the OMC and their affiliated hospitals for Olmsted County residents; by comparison, the Mayo Clinic warehouse currently uses only billing data from Mayo Clinic in Rochester for all patients regardless of where they live. The primary focus of this paper is the REP cost data warehouse.

Data are extracted from the institutions’ financial decision support systems (DSSs), which combine data from many sources to support practice analytics. The data include billing services, charges, diagnoses, patient demographic characteristics, payer information, individual provider information, and, for one institution, the internal costs. The advantage of using DSSs is that all data are already linked within the two institutions at the patient level, reducing the potential for errors when combining various patient, provider, charge, diagnosis, and billing files for professional and hospital services. The REP infrastructure performs the higher level linkage of patients across institutions [11]. Mayo Clinic DSS data are stored in an enterprise data warehouse; OMC DSS data are stored in the epidemiology program’s database (Sybase; SAP SE). In order to protect business-sensitive information, no internal costs or insurance information from the two institutions are included in the data warehouse and access to the OMC source data is limited to staff who are affiliated with REP. Investigators are prohibited from comparing the two institutions.

**Fig. 1:**
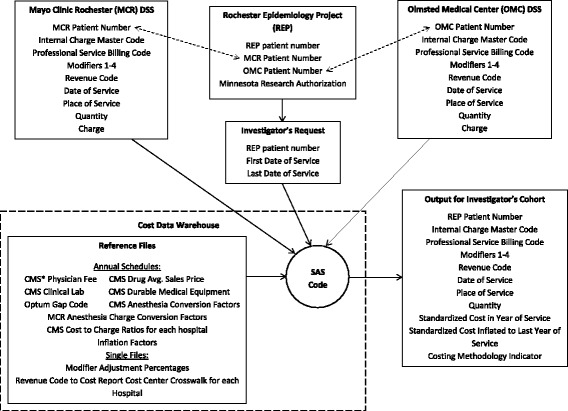
Cost Data Warehouse, Data Sources, and Output. CMS, Centers for Medicare and Medicaid Services

Changes in one institution’s DSS and adoption of a DSS by the other institution allowed us the opportunity to revisit the costing process and structure that had been in effect for OCHEUD for many years. We updated the programming code to refine the costing process and eliminate standardized cost data storage. We also distinguished between the REP and MCR populations by creating two warehouse names. The new warehouse requires no new storage space, is easier to maintain and to expand to other providers, and produces standardized costs for a requested cohort much faster. The relationship between the REP cost data warehouse, source files, and REP with the data fields used by the warehouse is shown in Fig. 1.

We have found that at a minimum, we need the following DSS data fields for each service: patient identifier, date of service, internal charge master code, the Healthcare Common Procedure Coding System (HCPCS) or Current Procedural Terminology Fourth Edition (CPT-4) code, all CPT-4 code modifiers, place of service (hospital vs clinic), quantity, charge, uniform billing (UB) revenue code, and a *final billed* indicator that refers to the final claim submission.

### Costing algorithm

Our costing algorithm is a hybrid of two methods: one for professional services billed to all payers on the Centers for Medicare and Medicaid Services (CMS) 1500 form and one for hospital services billed to all payers on the UB04 or CMS 1450 form. Details about how we handled special circumstances as well as sample SAS code are provided in Additional files 1 and 2, Additional files 3 and 4: Tables S1 and S2.

Professional services identified with either HCPCS or CPT-4 codes are assigned standardized costs by using national reimbursement amounts from the appropriate Medicare physician, clinical laboratory, Medicare part B drug average sales price, and durable medical equipment, prosthetics, orthotics, and supplies fee schedules. Services without an assigned Medicare fee, most of which are carrier-priced, are assigned a gap code fee from The Complete RBRVS Annual Data File (Optum360, Inc) [12]. These Medicare and Optum360 fees are based on relative value units (RVUs) that estimate relative resource utilization; although these fees represent third-party payer costs, they estimate relative provider costs as well. The sources of these reference files are listed in Table 1. Assigned costs are multiplied by quantity and then adjusted by appropriate modifier percentages (Table 2). A zero charge results in a zero cost. If a service has a negative charge and quantity reflecting a billing correction, it is assigned a negative cost.

**Table 1:** Reference Files

File	Source	File Name
Physician Services	https://www.cms.gov/Medicare/Medicare-Fee-for-Service-Payment/ClinicalLabFeeSched/Clinical-Laboratory-Fee-Schedule-Files.html http://www.cms.gov/Outreach-and-Education/Medicare-Learning-Network-MLN/MLNProducts/downloads/How_to_MPFS_Booklet_ICN901344.pdf	PPRVU*YY*_V*MMDD* csv, txt, or xlsx RVUPUF*YY.*docx
Clinical laboratory	http://www.cms.gov/Medicare/Medicare-Fee-for-Service-Payment/ClinicalLabFeeSched/clinlab.html	CLAB*YYYY*.EffJan1.Full **plus** CLAB*YYYY*.Eff*MMM*.*X*Codes if available csv, txt, or xlsx
DMEPOS	http://www.cms.gov/Medicare/Medicare-Fee-for-Service-Payment/DMEPOSFeeSched/DMEPOS-Fee-Schedule.html	DME*YYYY MMM* csv, txt, or xlsx
Parenteral and enteral nutrition items and services	http://www.cms.gov/Medicare/Medicare-Fee-for-Service-Payment/DMEPOSFeeSched/DMEPOS-Fee-Schedule.html	DME*YYYY MMM* PEN PUF csv, txt, or xlsx
Part B drug average sales price	http://www.cms.gov/Medicare/Medicare-Fee-for-Service-Part-B-Drugs/McrPartBDrugAvgSalesPrice/index.html	ASP pricing files csv or xlsx
Gap codes	https://www.optum360coding.com/Category/100037/100197/ (Optum360, Inc)	
Modifiers	https://www.wpsgha.com/wps/portal/mac/site/claims/guides-and-resources/modifiers/	By modifier
Anesthesia conversion factors	http://www.cms.gov/Center/Provider-Type/Anesthesiologists-Center.html	Conversion factors
Anesthesia base units	http://www.cms.gov/Center/Provider-Type/Anesthesiologists-Center.html	Base units
Cost to charge ratios^a^	ResDAC: http://www.resdac.org/media/calculating-cost-cost-charge-ratios	
UB revenue code by line	Internal list mapped for each institution	
Inflation factors	http://www.bea.gov/iTable/iTable.cfm?ReqID=9&step=1#reqid=9&step=3&isuri=1&903=13	Table 1.1.9. Implicit Price Deflators for Gross Domestic Product
Impute rates	Internally calculated on a year-to-date basis for every service year for each institution	

*Abbreviations*: *DMEPOS* Durable medical equipment, prosthetics/orthotics and supplies, *ResDAC* Research Data Assistance Center, *UB* uniform billing

^a^Cost to charge ratios are calculated from cost center costs and charges

**Table 2:** Modifiers That Affect Cost

Modifier	Type of Service	Definition	Impact
50	Physician fee and gap schedules	Bilateral procedure	Increase to 150% of fee/cost
51	Physician fee and gap schedules	Multiple procedure	Reduce to 50% of fee/cost
62	Physician fee and gap schedules	Cosurgeon	Reduce to 62.5% of fee/cost
AS, 80, 81, 82	Physician fee and gap schedules	Surgery assistant	Reduce to 16% of fee/cost
QK	Anesthesia	Medical direction by a physician of 2, 3, or 4 concurrent anesthesia procedures	Reduce to 50% of fee/cost
QX	Anesthesia	CRNA/AA service with medical direction by a physician	Reduce to 50% of fee/cost
QY	Anesthesia	Medical direction of one CRNA/AA by an anesthesiologist	Reduce to 50% of fee/cost
P1	Anesthesia	Healthy patient	Add 0 units to base
P2	Anesthesia	Patient with mild systemic disease	Add 0 units to base
P3	Anesthesia	Patient with severe systemic disease	Add 1 unit to base
P4	Anesthesia	Patient with severe systemic disease that is a constant threat to life	Add 2 units to base
P5	Anesthesia	Moribund patient who is not expected to survive without the operation	Add 3 units to base
P6	Anesthesia	A patient who has been declared “brain dead” and whose organs are being removed for donor purposes	Add 0 units to base

*Abbreviations*: *AA* anesthesiologist’s assistance, *CRNA* certified registered nurse anesthetist

The Physician Fee Schedule (PFS) includes three types of RVUs—for work, practice expense, and malpractice expense. The RVUs are summed and then multiplied by a conversion factor. The PFS and gap schedules contain different practice expense RVUs for many services, depending on whether the service was performed in a facility (e.g., hospital) or a nonfacility (or clinic) setting, because the allowable practice expense for the physician is lower where the facility can bill separately. Therefore, the place of service must be considered when determining the appropriate fee. PFS and gap schedules also include separate fees for some services among three different modifiers: 26 (or professional component), TC (technical component), and 53 (or discontinued procedure), so services with one of these modifiers in any of the four possible CPT-4 modifier fields must be matched to the fee schedules on both CPT-4 and modifier.

Anesthesia costing is more complex. Professional anesthesia reimbursement is based on time spent for a procedure, so there are no set fees for the CPT-4 codes. Three types of units—base, time, and physical status—are summed and then multiplied by a conversion factor. The base units are determined by the CPT-4 code that indicates the type of surgery and anesthesia. Time units are equal to the number of minutes divided by 15, then rounded to 0.1. Physical status units are determined by the American Society of Anesthesiologists physical status modifiers (ie, P1–P6) (Table 2), which represent a patient’s fitness before surgery. Finally, the resulting cost may be decreased by 50% when certain modifiers (listed in Table 2) indicate that more than one person performed the service, such as a physician who supervised an assistant or nurse anesthetist. The conversion factors are listed in the CMS file by locality; we use the national average value for each year. Alternatively, when billing conversion factors are available for the services in the DSS, charges can be divided by the billing conversion factors and multiplied by Medicare conversion factors to obtain the same result.

**Fig. 2:**
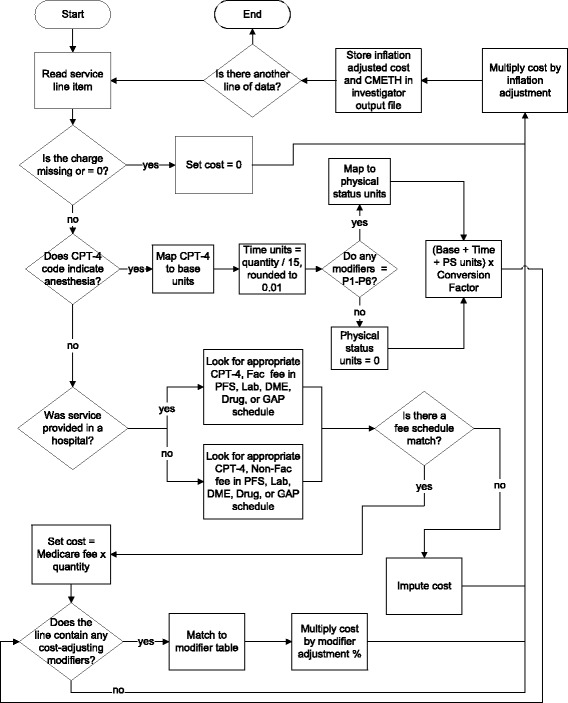
Professional Services Cost Algorithm. CMETH indicates costing methodology; CPT-4, Current Procedural Terminology Fourth Edition; DME, durable medical equipment; PFS, Physician Fee Schedule

Any nonanesthesia professional services that do not map to a fee schedule are assigned an imputed cost by multiplying the charge by an average professional service cost to charge ratio (CCR). Figure 2 illustrates the decision process for all professional services.

Since Medicare reimbursement for hospital services is based on a prospective payment, it cannot be used to create standardized costs for individual inpatient and outpatient services, such as supplies or use of an operating room. Therefore, we use CCRs from the Medicare cost report to convert charges for individual services into standardized costs. The Research Data Assistance Center provides excellent instruction for how to obtain the CCRs, so we do not go into those details here [13]. The challenge lies in what to do with the ratios.

Every hospital’s cost report contains hospital total costs and charges and a set of costs and charges aligned with various cost centers that can be used to calculate hospital level and cost center level CCRs. Although the Healthcare Cost and Utilization Project [14] provides hospital-level ratios for charge-to-cost conversion, we use cost center level CCRs to provide more accurate service level costs [2, 15]. There are two ways to map these CCRs to individual services: 1) match on the internal cost or revenue center that was used to assign the charges and costs to the cost report cost center and 2) use UB revenue codes, which are used by most researchers and the University HealthSystem Consortium [16]. For such services as room and board, the result is the same. We opted to use revenue codes since internal cost or revenue center information was not available for one institution and it was difficult to maintain the mappings for the other institution.

We created a crosswalk of UB revenue codes to cost report cost centers for each institution. When we did not have a good match, we used the hospital average CCR. Table 3 contains a sample from one crosswalk. Medicare occasionally creates new cost centers that need to be considered during our annual updates (e.g., computed tomography and magnetic resonance imaging were split from Diagnostic Radiology in 2011). We found that some CCRs varied between the two hospitals of Mayo Clinic and varied over time mainly because of changes in cost report preparation. We addressed this variation by combining the costs and charges of these two Mayo hospitals and then using 3 consecutive years of costs and charges to create 3-year moving average ratios. We also use moving average ratios for the OMC hospital. Since cost report availability is delayed by about 1 year, we continue to use the preceding year’s ratios for the current year’s services until a new report is available.

**Table 3:** Sample UB Revenue Code to Cost Report Cost Center Crosswalk

UB Revenue Codes^a^	Revenue Code Descriptions	Cost Report Cost Centers
0115–0122	Room and Board	Adults and Pediatrics (General Routine Care)
0171–0173	Nursery	Nursery
0200	Intensive Care Unit	Intensive Care Unit
0250–0258	Pharmacy and Drugs	Drugs Charged to Patients
0260–0264	Intravenous Therapy	Drugs Charged to Patients
0270–0279	Supplies	Medical Supplies Charged to Patients
0290–0299	Medical Equipment	Medical Supplies Charged to Patients
0300–0319	Laboratory and Pathology	Laboratory
0320–0324	Diagnostic X-Ray	Radiology-Diagnostic
0340–0343	Nuclear Medicine	Radiology-Diagnostic
0350–0359	Computed Tomography (CT) Scan	Computed Tomography (CT) Scan
0360	Operating Room	Operating Room
0370	Anesthesiology	Anesthesiology
0390–0391	Blood	Blood
0401–0403	Mammography and Ultrasonography	Radiology-Diagnostic
0410–0413	Respiratory and Hyperbaric Therapy	Respiratory Therapy
0420–0429	Physical Therapy	Physical Therapy

^a^Assignment depends on the revenue codes appearing in the hospital’s decision support system data and the Cost Centers appearing in that hospital’s Medicare Cost Report

The resulting assigned costs for all services are adjusted for inflation using the gross domestic product (GDP) Implicit Price Deflator [17, 18] to the final year of an investigator’s study period as the last step in the costing algorithm.

### Warehouse structure

The warehouse consists of SAS software version 9.4 (SAS Institute Inc.) code and a set of reference files. All professional costing fee schedules are stacked into one file containing columns for CPT-4 and HCPCS codes (listed in a sole column), modifier, year, nonfacility fee, facility fee, and source fee schedule. They are arranged first by year, then code, and then modifier for every code. Since only the PFS and gap schedule have separate fees for some facility and nonfacility services, the same fee often appears in both columns. This one file is used for both institutions and currently contains over 246,000 lines for 2003 through 2015.

Three additional reference files are needed for costing professional services: modifiers with associated cost adjustment percentages, anesthesia conversion factors by year, and imputation rates by year and institution. Imputation rates are used to estimate a cost where a professional service cannot be mapped to a non-zero fee. An annual rate is created by running the costing algorithm against all of the data for a service year. We then calculate the ratio of the total assigned fees to the total charges for all services (excluding zero-charge services) to create an average standardized cost-to-charge imputation rate that can be multiplied by the imputed services’ charges.

The two institutions’ hospital costing reference files each contain the CCRs, year, and UB revenue codes arranged first by year and then by revenue code. The creation of the CCR files is a manual process because it requires judgment about which cost center costs and charges need to be combined to create ratios (eg, operating room, recovery room) and which ratios are mapped to the various UB revenue codes. The inflation file is organized first by the year in which the service occurred and then by the target year to which costs are to be inflated, with an inflation index consisting of the GDP of the target year divided by the GDP of the service year for every combination of years.

When we reach a new calendar year, we download the new year of CMS files, purchase the new Optum Gap Code schedule, download inflation factors from the Bureau of Labor Statistics, access the institutions’ cost reports to create new cost to charge ratios, and apply our SAS code to update the reference files. We apply the costing algorithm to assign uninflated and inflated costs and an indicator of the costing methodology (i.e., PFS, laboratory, DME (durable medical equipment), drug, gap, anesthesia, CCR, zero charge, or impute) to every service in the new time period and run quality control. Our quality control code identifies issues such as UB revenue codes in the data that need to be added to the CCR reference file. The quality control outputs a list of all CPT-4 codes that are imputed so we can determine whether a reference file adjustment is needed. Our quality control also looks at trends in charges and costs over time and checks the proportions of the various cost methods in the data. Finally, samples of data are manually validated to ensure accuracy.

Expansion of the cost data warehouse to a new provider requires primarily the creation of a new set of provider-specific cost to charge ratios and a crosswalk of the CCRs to cost report cost centers. The quality control code and sample manual validation will highlight any billing anomalies that need to be accommodated by adjusting the costing algorithm.

When an investigator submits a request for data by providing patient identification numbers and the ranges of dates of service, our algorithm first checks for both centers’ institutional review board approval for the study and the appropriate Minnesota Research Authorization status [19, 20] for every patient. It then extracts all available service line data for the specified date ranges and patients from the enterprise data warehouse and REP database and applies the costing algorithm to create inflation- adjusted standardized costs. Investigators always receive services and standardized costs based on the most up-to-date source data, since we store no versions of data sets with cost.

The final data set provided to investigators includes a list of services with inflation-adjusted standardized costs and cost methods for each service, identifiable by HCPCS (or CPT-4) code or UB revenue code, or both; charge master code; patient identifier; date of service; and place of service. We include service description and service line level (vs claim or encounter level) diagnoses. Data for each hospitalization and billing episode are available in a separate file so the investigator has easy access to such information as patient demographic characteristics, admission source, and discharge disposition. To maintain business sensitivity of the data, only the standardized costs are provided.

## Results

To better understand the costing process, we present the distribution of cost method by lines of data for Mayo Clinic from 2003 through 2014 (Table 4). Three numbers stand out. The first is the low level—less than 1%—of professional service lines that were not matched to fee schedules and were imputed. The two other interesting categories are those with zero charges. A sample of Mayo Clinic’s zero-charge services provided in the clinic showed that they were primarily unlisted procedures (CPT-4 codes often ending in “99”) that carry no set fee and are reimbursed only on appeal to the CMS carrier; bundled visits such as surgery follow-up and obstetric care that are part of a global payment; and reporting and measurement codes. A sample of hospital zero-charge services showed that 99.7% were “pharmacy labor” lines where the DSS contains separate lines for pharmaceutical preparation labor and the drug itself for cost accounting purposes, but the labor portion has no associated charge. This latter case may not be common among providers, but again, it is shared as another example of how the underlying source data must be understood.

**Table 4:** Mayo Clinic Cost Methods, 2003–2015

Cost Method	% of Total Lines	% of Professional or Hospital
Professional services	48.6	
Anesthesia		0.8
Clinical laboratory		28.0
Drugs		1.7
Durable medical equipment		<0.1
Gap		4.5
Header		3.3
Impute		0.8
Physician Fee Schedule		32.8
Zero charge		28.1
Hospital services	51.4	
Cost to charge ratio		73.4
Zero charge		26.6

## Discussion

Healthcare cost data used for research break into several broad categories. Some researchers share actual internal costs, but the costing method and degree of health service coverage vary. Intermountain Healthcare investigators have published studies using data from an activity- based cost accounting system, but it only contains hospital costs [21, 22]. Filice et al. [23] used internal costs from a Department of Veterans Affairs cost accounting system, but the department does not share the confidentiality concerns of nongovernment providers. Abbott and Meara [24] used actual, fully loaded (ie, incorporates fixed costs, administrative overhead, and more) facility costs but approximated actual professional costs by using physician specialty–specific CCRs multiplied by professional charges. Abbott and Meara [24] and Chang et al. [25] state that they used costs derived from institutional financial reports; the types of costs are enumerated, but the cost method is not completely clear. An advantage of using internal costs is that they are often created and validated in accounting systems—although many providers prohibit investigators from disclosing costs considered to be business-sensitive.

Time-based versions of internal costs have been used by several researchers [26–28]. Often, the actual costs of supplies and drugs are included, as well as estimates of space, administrative overhead, and equipment depreciation. Sometimes, data are supplemented with estimates and costs from secondary sources [29]. These time-based costs tend to be study specific, and the extent to which these costs are fully loaded is often unclear.

A third category of costs uses a combination of actual costs, Medicare reimbursement, or reimbursement tied to Medicare weights. Schousboe et al. [30, 31] have recommended a combination of Medicare professional and CMS Hospital Outpatient Prospective Payment System reimbursement plus hospital inpatient costs based on diagnosis-related group (DRG) weights. Ritzwoller et al. [32] seemed to modify the DRG facility costs by adjusting for certain professional services. However, the use of any type of DRG-based inpatient cost prohibits measurement of the cost impact of changes in specific inpatient service utilization or length of stay that would be possible with microcosting. Mabry et al. [33] added physician and nurse anesthetist costs and charges and removed those of particular services in which the investigators were not interested, modifying Medicare cost report data to create new CCRs to multiply by the charges of several institutions. This methodology would provide a more consistent approach to professional and hospital standardized costs than we have presented, but it would be difficult to implement beyond a study-specific scale. Bittl et al. [34] used a combination of actual nurse salaries and supply costs plus Medicare fees for physician reimbursement. They did not include facility costs because their study’s patients were not hospitalized.

Our costing algorithm is based on well-understood and widely accepted methodologies [8]. The cost data warehouse has all of the strengths of administrative data for estimating healthcare costs listed by Riley [2]. It avoids three of Riley’s listed limitations: Our patient identifiers do not change over time, we do not have difficulty using data from our accounting systems, and the raw data already are, or can easily be, grouped into visits. Our cost data warehouse was created and is maintained by expert staff familiar with the billing and DSSs, as well as Medicare reimbursement rules, similar to the recommendations of Meenan et al. [35]. The staff has excellent connections to the institutions’ finance colleagues when questions arise. This expertise is particularly important when dealing with CCRs, which, as Riley pointed out, can be complex and difficult to use. As a result, investigators who lack this degree of knowledge can still conduct cost studies. Our identified data can be linked to patient health records and patient registries, and because the data cover both institutions, patient-level utilization and costs can be tracked longitudinally. The data are not limited to patients of a certain age or insurance coverage. Inclusion of professional costs allows the analysis of outpatient care provided in a clinic setting; inclusion of service level costs for the hospitals allows analysis of how utilization can drive cost. The individual service and procedure costs can be aggregated into clinically meaningful categories with tools such as Berenson-Eggers Type of Service [36] and the Healthcare Cost and Utilization Project’s Clinical Classifications Software for Services and Procedures [37]. The design of the data warehouse allows for easy updates, does not require huge storage space, can be modified as data sources change, and can be expanded to include other providers.

The first publication using our cost data warehouse was in 1999 [38]. The warehouse structure and algorithm have since been updated. Recent publications using this data warehouse cover a wide range of procedures, conditions, and patient care cycles [39–44]. These studies’ results are often of interest to other providers, academic researchers, and policy makers.

There are some limitations to using our cost data warehouse. It can be used only to approximate direct healthcare costs. It contains some services (eg, dental, optical) where the cost of care for most patients is incomplete because they receive the services outside of Mayo Clinic and OMC. However, investigators are free to exclude these limited services from their analyses. Services that are not billed, such as follow-up phone calls, but might be available in an activity-based costing system are not included.

Outpatient drugs obtained from a retail pharmacy that might appear in claims data are also not included. Currently, no facility services for long-term care or skilled nursing care or any home health agency services are captured in the data warehouse. Studies may not be able to incorporate the cost of all healthcare provided to patients, especially referral patients. Finally, due to billing rules and the reliance on CMS fee schedules and cost reports, this costing methodology is only applicable for United States providers.

**Fig. 3:**
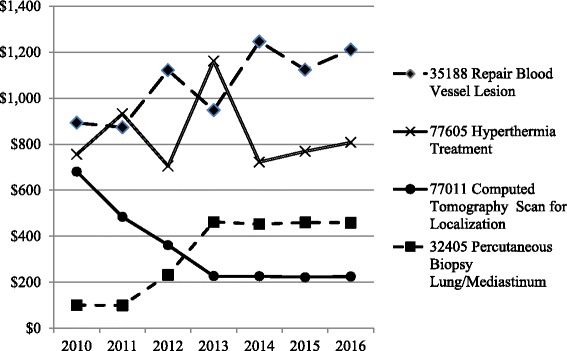
Medicare Fee Variation for Selected CPT-4 Codes Over Time. Bx indicates biopsy; CPT-4, Current Procedural Terminology Fourth Edition; CT, computed tomography

Perhaps the greatest limitation is one common to nearly all sources of standardized costs: the inability to easily examine cost trends. Both professional and hospital standardized costs can vary over time because of factors unrelated to inflation, such as Medicare RVU and conversion factor adjustments, changes in Medicare cost report structure, and changes in how costs and charges are assigned by the institutions to cost report cost centers. Figure 3 illustrates four CPT-4 codes for which the PFS national fees have dramatically changed over 5 years. These variations can make it difficult to discern whether changes in total costs over time are due to changes in utilization or changes in unit costs. However, our costing algorithm can be applied in a limited manner to map all costs to those in effect during a study’s final year, thereby eliminating this issue [45].

## Conclusion

After its development, a provider-based, standardized healthcare cost data warehouse can be maintained easily. It also can be expanded to include other providers, increasing the size of the potential study population and facilitating more powerful, detailed, bottom-up cost studies. We believe that our standardized costs are useful both for providing reasonable and understandable total cost estimates and for describing relative costs. The data warehouse automates the process of generating standardized costs, and therefore, individual investigators, who may not have sufficient knowledge about administrative data, do not have to try to create their own standardized costs on a project-by-project basis.

## Additional files

Additional file 1: Special Costing Circumstances. (DOCX 21 kb)

Additional file 2: Cost Data Warehouse SAS Program for Costing Algorithm. (DOCX 28 kb)

Additional file 3: Table S1. Billing Data Structure. (DOCX 21 kb)

Additional file 4: Table S2. Reference Files Structure. (DOCX 22 kb)

## Abbreviations

CCR: Cost to charge ratio
CMS: Centers for Medicare and Medicaid Services
CPT-4: Current Procedural Terminology Fourth Edition
DRG: Diagnosis-related group
DSS: Decision support system
GDP: Gross domestic product
HCPCS: Healthcare Common Procedure Coding System
OCHEUD: Olmsted County Healthcare Expenditure and Utilization Database
PFS: Physician Fee Schedule
REP: Rochester Epidemiology Project
RVU: Relative value unit
TC: Technical component
UB: Uniform billing
