# Supplementary material for: Developing a standardized healthcare cost data warehouse
Source: BMC Health Serv Res. 2017 Jun 12;17:396. doi: 10.1186/s12913-017-2327-8 (PMC5469019; doi:10.1186/s12913-017-2327-8)
Supplement: Supplementary file 1 — Special Costing Circumstances. (DOCX 21 kb) [file 12913_2017_2327_MOESM1_ESM.docx]

**Special Costing Circumstances**

**Distinguishing between professional and hospital-billed services**

The first step is to correctly identify which services are in each category.

One institution’s decision support system (DSS) contains separate fields for charges billed out of the hospital and charges for all professional and nonhospital technical services billed out of the clinic. The other institution’s DSS begins the patient encounter numbers billed out of the clinic with a “C” and those billed out of the hospital with an “H.” These encounters mostly align with professional and hospital services, but the alignment is not completely straightforward because some professional and hospital services, such as radiologic examinations, are combined on 1 line billed out of the hospital. Charges for these services, identified by the charge master code, are split into assigned proportions of professional and hospital components, for the 2 costing methods to be applied. Finally, we use the *hospital costing method* for any service with a technical component (TC) modifier plus hospital inpatient or outpatient place of service, and we use the *professional method* for any service with a TC modifier plus clinic place of service.

# Services with a quantity of zero

In an institution’s DSS, some services, such as having an assistant in a surgical procedure, have a quantity of zero, thus an exception to multiplying by quantity is built into the code when a surgery assistant modifier (AS or 80) is present.

# Medicare Physician Fee Schedule details

A Medicare exception for 2007 and 2008 requires that a budget neutrality factor be multiplied by only the work relative value units (RVUs) before the 3 RVUs are summed (1,2).

During the years when Medicare is in the midst of adjusting RVU weights, the PFS includes 2 different practice expense RVUs, *transitional* and *fully implemented*. Our finance colleagues have advised us to always use the transitional values whenever an option exists, since the fully implemented values reflect fees expected to be in effect in a later year. Although the schedules are published approximately 4 times annually, we use the most recent, or last, version for each year unless it is a schedule (2003-2013) with roughly 20% conversion factor reductions for sustainable growth rate adjustment implementation.

# Current Procedural Terminology Fourth Edition Code Modifiers

One of our institutions includes a provider-based clinic that bills some professional services and their associated technical components out of the hospital. We have chosen to treat these services as clinic services instead of hospital outpatient services, to be consistent with the other clinic services in the cost data warehouse. Many of these services appear in the DSS data with professional component (PC; similar to 26) and TC modifiers but have only global lines in the fee schedule. Our costing algorithm defaults to the global fee for the 2 portions, thereby doubling the standardized cost. We flag those modifier services that default to a global fee and then clean up the duplicate lines at the level of the much smaller investigator data sets.

# Professional Geographic Pricing Cost Index

Actual Medicare reimbursement includes a factor in the Physician Fee Schedule calculation to adjust for geographic location (Geographic Pricing Cost Index). We chose not to make this adjustment because we want to approximate national reimbursement.

# Hospital Wage Index

Wage indices are available to adjust the resulting standardized costs for hospital services to national levels. The University HealthSystem Consortium has performed extensive analysis to estimate the proportion of labor in the various cost centers and only applies the wage index adjustment to the labor proportions. We do not have that detailed information for our hospitals, so we did not include wage index adjustment.

# Reference Fee Schedule

The various fee schedules are stacked in order of year, then Current Procedural Terminology Fourth Edition (CPT-4) or Healthcare Common Procedure Coding System (HCPCS) code, and then 26, 53, and TC modifiers for each code when applicable. The Physician Fee Schedule is first checked for nonzero fees for a CPT-4 or HCPCS code and modifier combination. If the code is not found in the Physician Fee Schedule or the fees are equal to zero, then the Lab schedule is checked. The other Medicare schedules follow, with the gap schedule checked last. Sometimes, the global fee appears in 1 schedule and the modifier fees in another schedule. If no nonzero fees are found in any schedules for a CPT-4/ HCPCS code, then “$0.00” is placed on the line as the standardized cost. The last schedule’s fee used to create the standardized cost for a particular CPT-4/HCPCS code in the reference file is listed in a source fee schedule column. When the costing algorithm is applied to this service found in a data set with a nonzero charge, the standardized nonzero cost is multiplied by the quantity and adjusted by modifier percentages. If the reference file contains “$0.00,” then a standardized cost is imputed.

**References**

1. Centers for Medicare and Medicaid Services. Physician Fee Schedule [Internet] [2007; cited 2015 Aug 21]. Available from: <http://www.cms.gov/Medicare/Medicare-Fee-for-Service-> Payment/PhysicianFeeSched/PFS-Relative-Value-Files- Items/CMS1200161.html?DLPage=5&DLSort=0&DLSortDir=descending.
2. Centers for Medicare and Medicaid Services. Physician Fee Schedule [Internet] [2008; cited 2015 Aug 21]. Available from: <http://www.cms.gov/Medicare/Medicare-Fee-for-Service-> Payment/PhysicianFeeSched/PFS-Relative-Value-Files- Items/CMS1209430.html?DLPage=4&DLSort=0&DLSortDir=descending.
