# Supplementary material for: Developing a standardized healthcare cost data warehouse
Source: BMC Health Serv Res. 2017 Jun 12;17:396. doi: 10.1186/s12913-017-2327-8 (PMC5469019; doi:10.1186/s12913-017-2327-8)
Supplement: Supplementary file 2 — Cost Data Warehouse SAS Program for Costing Algorithm. (DOCX 28 kb) [file 12913_2017_2327_MOESM2_ESM.docx]

**Cost Data Warehouse SAS Program for Applying Cost**

**Original program (example)**

*-------------------------------------------------------------------------*

|Authors: Stephanie Anderson and Megan Reinalda

*-------------------------------------------------------------------------*;

%include "/projects/hcpr/activity/s112837.CDW/production/saspgm/onthefly/cost_dss_data.sas";

%include "/projects/hcpr/activity/s112837.CDW/production/saspgm/onthefly/inflate.sas";

**********************************************************************;

** Pull Billing Data **;

** See Table 1 Billing Data Structure below **;

**********************************************************************;

options symbolgen mlogic mprint;

%cost_dss_data(indsn=out.dss_edt, costeddsn=costed_cohort);

**************************************************;

** Inflate data **;

**************************************************;

%inflate(dsn=costed_cohort, inflateyear=2014);

data out.costed;

retain encnbr clinic dateserv cpt4 cpt4hdr cpt4mod cpt4mod2 cpt4mod3 cpt4mod4 feederky orgvolum location

ubcode year billstat ldx1 ldx2 ldx3 ldx4 cost_parta cost_partb cmeth_parta

cmeth_partb cost_part: total_cost:

set costed_cohort;

run;

**Code to apply costing**

*-------------------------------------------------------------------------*

|S112837: CDW

|Program Created: 05/10/2013

|Program Updated: 05/10/2013

|

|Authors: Stephanie Anderson and Megan Reinalda

|

|Input: A SAS dataset of DSS data

|Output: Costed DSS data, costed with the CDW costing algorithm

*-------------------------------------------------------------------------*;

%macro cost_dss_data(indsn=edt, costeddsn=costed);

*-------------------------------------------------------------------------*

|See Table 2 Reference Files below

*-------------------------------------------------------------------------*;

libname ref '/projects/hcpr/activity/s112837.CDW/production/ref_master';

*-------------------------------------------------------------------------*

|Determine Costing Method

|1. Zero Charge

|2. Cost-to-Charge Ratio

|3. Header Schedule

|4. CPT Schedule

*-------------------------------------------------------------------------*;

data &indsn;

set &indsn;

rename fyear=year cpt=cpt4;

run;

data _zerochg _ubcode _header _cpt4;

set &indsn;

if actchrg in (0,.) then output _zerochg;

else if cpt4 in ('','00000') then output _ubcode;

else if cpt4hdr = 'H' then output _header;

else if partbchg in (0,.) and location=’Hospital’ then output _ubcode;

else output _cpt4;

run;

*-------------------------------------------------------------------------*

|CPT4

*-------------------------------------------------------------------------*;

data _cpt4;

length mod $2 cpt4 $5;

set _cpt4;

if cpt4mod in ('26' 'TC') then mod=cpt4mod;

else if cpt4mod2 in ('26' 'TC') then mod=cpt4mod2;

else if cpt4mod3 in ('26' 'TC') then mod=cpt4mod3;

else if cpt4mod4 in ('26' 'TC') then mod=cpt4mod4;

run;

*Index data;

proc sql noprint;

create index cptmodyr on _cpt4 (cpt4, mod, year);

quit;

proc sql noprint;

create table ref_feesched as select * from ref.feesched;

create index cptmodyr on ref_feesched (cpt4, mod, year);

quit;

data _cpt4costed _cpt4notmapped;

merge _cpt4(in=in1) ref_feesched(in=in2);

by cpt4 mod year;

if in1;

if in1 and in2 then output _cpt4costed;

else output _cpt4notmapped;

run;

/* _cpt4notmapped is a quality check. This should be empty. */

proc datasets lib=work nolist;

delete _cpt4;

run; quit;

*-------------------------------------------------------------------------*

|Apply additional modifiers that affect payment:

|AS Assistant at surgery 16%

|50 Bilateral 150%

|51 Multiple procedure 50%

|62 Co-surgery 62.50%

|80 Assistant surgeon 16%

|81 Assistant surgeon 16%

|82 Assistant surgeon 16%

*-------------------------------------------------------------------------*;

data _cpt4costed _costccr;

set _cpt4costed(rename=(cmeth=cmeth_partb));

*If a modifier causes a fee change, then set adjustment factor, else let adjustment factor be 1 (so multiplication works later);

if cpt4mod = 'AS' or cpt4mod2 = 'AS' or cpt4mod3 = 'AS' or cpt4mod4 = 'AS' then adj1=0.16; else adj1=1;

if cpt4mod = '50' or cpt4mod2 = '50' or cpt4mod3 = '50' or cpt4mod4 = '50' then adj2=1.50; else adj2=1;

if cpt4mod = '51' or cpt4mod2 = '51' or cpt4mod3 = '51' or cpt4mod4 = '51' then adj3=0.50; else adj3=1;

if cpt4mod = '62' or cpt4mod2 = '62' or cpt4mod3 = '62' or cpt4mod4 = '62' then adj4=0.625; else adj4=1;

if cpt4mod = '80' or cpt4mod2 = '80' or cpt4mod3 = '80' or cpt4mod4 = '80' then adj5=0.16; else adj5=1;

if cpt4mod = '81' or cpt4mod2 = '81' or cpt4mod3 = '81' or cpt4mod4 = '81' then adj6=0.16; else adj6=1;

if cpt4mod = '82' or cpt4mod2 = '82' or cpt4mod3 = '82' or cpt4mod4 = '82' then adj7=0.16; else adj7=1;

*----------------------------------------------------------------------------------*

|Procedures performed in the hospital are assigned the facility fee.

|Procedures performed in the clinic are assigned the clinic fee.

|Our billing data includes a volume of 0 with modifiers AS and 80,

|so we do not multiply the fee by volume for those AS and 80 services.

*----------------------------------------------------------------------------------*;

if location=’Hospital’ then do;

if orgvolum ne 0 then cost_partb=feef*orgvolum*adj1*adj2*adj3*adj4*adj5*adj6*adj7;

else cost_partb=feef*adj1*adj2*adj3*adj4*adj5*adj6*adj7;

if partachg ^in (. 0) then output _costccr;

else output _cpt4costed;

end;

else do;

if orgvolum ne 0 then cost_partb=fee*orgvolum*adj1*adj2*adj3*adj4*adj5*adj6*adj7;

else cost_partb=fee*adj1*adj2*adj3*adj4*adj5*adj6*adj7;

output _cpt4costed;

end;

drop adj1-adj7 fee feef;

run;

*-------------------------------------------------------------------------*

|HEADER

*-------------------------------------------------------------------------*;

proc sql noprint;

create table ref_headersched as select * from ref.header;

create index feedyr on ref_headersched (feederky,year);

quit;

proc sql noprint;

create index feedyr on _header (feederky,year);

quit;

data _headercosted(rename=(cmeth=cmeth_partb)) _headernotmapped(drop=cmeth);

merge _header(in=in1) ref_headersched(in=in2);

by feederky year;

if in1;

if in1 and in2 then output _headercosted;

else output _headernotmapped;

run;

/* _headernotmapped is a quality check. This should be empty. */

data _headercosted;

set _headercosted;

cost_partb=fee*orgvolum;

cmeth_partb='H';

run;

*-------------------------------------------------------------------------*

|IMPUTE

*-------------------------------------------------------------------------*;

proc sql noprint;

create table ref_imputerate as select * from ref.imputerate;

create index year on ref_imputerate (year);

quit;

proc sql noprint;

create index year on _cpt4costed (year);

quit;

data _cpt4costed;

merge _cpt4costed(in=in1) ref_imputerate;

by year;

if in1;

if cost_partb=0 then do;

cost_partb=partbchg*imputeccr;

cmeth_partb='IMP';

end;

drop imputeccr;

run;

*-------------------------------------------------------------------------*

|CCR

*-------------------------------------------------------------------------*;

proc sql noprint;

create table ref_mayo_ccr as select * from ref.mayo_ccr;

create index ubyr on ref_mayo_ccr (ubcode,year);

quit;

data _ubcode;

set _ubcode _costccr _costccr2;

run;

proc sql noprint;

create index ubyr on _ubcode (ubcode,year);

quit;

proc sort data = ref_mayo_ccr nodupkey;

by cmeth ubcode year ccr;

run;

data _ubcodecosted(rename=(cmeth=cmeth_parta));

merge _ubcode(in=in1) ref_mayo_ccr(in=in2);

by ubcode year;

if in1;

if partachg^=0 then cost_parta=partachg*ccr;

else cost_parta=0;

drop ccr;

run;

*-------------------------------------------------------------------------*

|Zero Charges

*-------------------------------------------------------------------------*;

data _zerocosted;

length cmeth_parta cmeth_partb $3;

set _zerochg;

cost_parta=0;

cost_partb=0;

cmeth_parta='Z';

cmeth_partb='Z';

run;

*-------------------------------------------------------------------------*

|Combine costed files

*-------------------------------------------------------------------------*;

data &costeddsn;

set _cpt4costed _headercosted _ubcodecosted _zerocosted;

label cost_parta ='Standardized Cost (Part A)'

cost_partb ='Standardized Cost (Part B)'

clinic='Clinic Number'

orgvolum='ORIGINAL_VOLUME'

cmeth_parta='Costing Method (Part A)'

cmeth_partb='Costing Method (Part B)';

format cost_parta cost_partb dollar10.2

dateserv mmddyy10.;

drop status descript mod;

run;

*-------------------------------------------------------------------------*

|Clean-up work library

*-------------------------------------------------------------------------*;

proc datasets lib=work nolist;

delete _: ref_:;

run;quit;

libname ref clear;

%mend;

**Inflation macro**

%macro inflate(dsn=, inflateyear=);

libname ref '/projects/hcpr/activity/s112837.CDW/production/ref_master';

data _ref_inflate(keep=year inflateyear index);

set ref.inflation(where=(inflateyear=&inflateyear));

run;

libname ref clear;

proc sort data=_ref_inflate;

by year;

run;

proc sql noprint;

create index year on &dsn (year);

quit;

data &dsn;

merge &dsn(in=in1) _ref_inflate(in=in2);

by year;

if in1;

cost_parta&inflateyear=cost_parta*index;

cost_partb&inflateyear=cost_partb*index;

total_cost&inflateyear=sum(of cost_parta&inflateyear cost_partb&inflateyear);

format cost_parta&inflateyear cost_partb&inflateyear total_cost&inflateyear dollar10.2;

label cost_parta&inflateyear = "Cost (Part A) inflated to &inflateyear dollars"

cost_partb&inflateyear = "Cost (Part B) inflated to &inflateyear dollars"

cost_parta = "Uninflated Cost (Part A)"

cost_partb = "Uninflated Cost (Part B)"

total_cost&inflateyear = "Total Cost inflated to &inflateyear dollars";

drop index inflateyear;

run;

proc datasets lib=work nolist;

delete _ref_inflate;

quit;

%mend inflate;
