# Supplementary material for: Developing a standardized healthcare cost data warehouse
Source: BMC Health Serv Res. 2017 Jun 12;17:396. doi: 10.1186/s12913-017-2327-8 (PMC5469019; doi:10.1186/s12913-017-2327-8)
Supplement: Supplementary file 3 — Billing Data Structure. (DOCX 21 kb) [file 12913_2017_2327_MOESM3_ESM.docx]

**Table S1.** Billing Data Structure

| **Field** | **Format** | | | **Description** |
| --- | --- | --- | --- | --- |
| clinic | Num | 8 |  | Medical record number |
| encnbr | Char | 20 |  | Billing encounter number |
| dateserv | Num | 8 | MMDDYY10. | Date of service |
| year | Num | 8 |  | Fiscal year |
| cpt4 | Char | 5 |  | CPT4 code |
| cpt4hdr | Char | 1 |  | CPT4 header flag ^a^ |
| cpt4mod | Char | 2 |  | CPT4 1^st^ modifier |
| cpt4mod2 | Char | 2 |  | CPT4 2^nd^ modifier |
| cpt4mod3 | Char | 2 |  | CPT4 3^rd^ modifier |
| cpt4mod4 | Char | 2 |  | CPT4 4^th^ modifier |
| feederky | Char | 30 |  | Charge master code |
| ubcode | Char | 4 |  | Uniform Billing revenue code |
| orgvolum | Num | 8 |  | Original volume |
| location | Char | 8 |  | Place of service |
| ldx1 | Char | 6 |  | 1^st^ line item diagnosis ^b^ |
| ldx2 | Char | 6 |  | 2^nd^ line item diagnosis |
| ldx3 | Char | 6 |  | 3^rd^ line item diagnosis |
| ldx4 | Char | 6 |  | 4^th^ line item diagnosis |
| actchrg | Num | 8 |  | Total charge |
| partachg | Num | 8 |  | Part a charge |
| partbchg | Num | 8 |  | Part b charge |

^a^CPT4 Header Flag indicates that the CPT4 service code represents more than one charge master code.

^b^The line item diagnoses (ICD-9 and ICD-10) are not used in the costing process, but are extracted from the billing data for the investigator’s use.
