# Supplementary material for: Developing a standardized healthcare cost data warehouse
Source: BMC Health Serv Res. 2017 Jun 12;17:396. doi: 10.1186/s12913-017-2327-8 (PMC5469019; doi:10.1186/s12913-017-2327-8)
Supplement: Supplementary file 4 — Reference Files Structure. (DOCX 22 kb) [file 12913_2017_2327_MOESM4_ESM.docx]

**Table S2.** Reference Files Structure

| **File / Field** | **Format** | | | **Description** |
| --- | --- | --- | --- | --- |
| **feesched** |  |  |  |  |
| cmeth | Char | 3 |  | Costing method |
| cpt4 | Char | 5 |  | CPT4 code |
| mod | Char | 2 |  | Modifier (26, 53, or TC) |
| status | Char | 1 |  | Status code |
| year | Num | 8 |  | Year |
| fee | Num | 8 | DOLLAR10.2 | Fee for service provided in clinic |
| feef | Num | 8 | DOLLAR10.2 | Fee for service provided in hospital |
|  |  |  |  |  |
| **header** |  |  |  |  |
| feederky | Char | 5 |  | Charge master code |
| fee | Num | 8 | DOLLAR10.2 | Fee |
| cmeth | Char | 3 |  | Costing method |
| year | Num | 8 |  | Year |
| descript | Char | 20 |  | Description |
|  |  |  |  |  |
| **imputerate** |  |  |  |  |
| imputeccr | Num | 8 |  | Impute rate for professional services |
| year | Num | 8 |  | Year |
|  |  |  |  |  |
| **mayoccr** |  |  |  |  |
| cmeth | Char | 3 |  | Costing method |
| ubcode | Char | 5 |  | Uniform Billing revenue code |
| year | Num | 8 |  | Year |
| ccr | Num | 8 |  | Cost-to-charge ratio for hospital services |
|  |  |  |  |  |
| **inflation** |  |  |  |  |
| year | Num | 8 |  | Year of service |
| inflateyear | Num | 8 |  | Year to which costs are to be inflated |
| year_gdp | Num | 8 |  | GDP in year of service |
| inflateyear_gdp | Num | 8 |  | GDP in inflateyear |
| index | Num | 8 |  | inflateyear_gdp / year_gdp |
